# Mechanisms of Action of Hematopoietic Transcription Factor PU.1 in Initiation of T-Cell Development

**DOI:** 10.3389/fimmu.2019.00228

**Published:** 2019-02-20

**Authors:** Ellen V. Rothenberg, Hiroyuki Hosokawa, Jonas Ungerbäck

**Affiliations:** Division of Biology and Biological Engineering, California Institute of Technology, Pasadena, CA, United States

**Keywords:** transcription factor, developmental gene regulation, chromatin, T lymphocyte development, thymus, gene network, cell signaling, hematopoiesis

## Abstract

PU.1 is an ETS-family transcription factor that plays a broad range of roles in hematopoiesis. A direct regulator of myeloid, dendritic-cell, and B cell functional programs, and a well-known antagonist of terminal erythroid cell differentiation, it is also expressed in the earliest stages of T-cell development of each cohort of intrathymic pro-T cells. Its expression in this context appears to give T-cell precursors initial, transient access to myeloid and dendritic cell developmental competence and therefore to represent a source of antagonism or delay of T-cell lineage commitment. However, it has remained uncertain until recently why T-cell development is also intensely dependent upon PU.1. Here, we review recent work that sheds light on the molecular biology of PU.1 action across the genome in pro-T cells and identifies the genes that depend on PU.1 for their correct regulation. This work indicates modes of chromatin engagement, pioneering, and cofactor recruitment (“coregulator theft”) by PU.1 as well as gene network interactions that not only affect specific target genes but also have system-wide regulatory consequences, amplifying the impact of PU.1 beyond its own direct binding targets. The genes directly regulated by PU.1 also suggest a far-reaching transformation of cell biology and signaling potential between the early stages of T-cell development when PU.1 is expressed and when it is silenced. These cell-biological functions can be important to distinguish fetal from adult T-cell development and have the potential to illuminate aspects of thymic function that have so far remained the most mysterious.

## Introduction

### PU.1 Expression in Precursors of T Cells

PU.1, encoded by the *Spi1* gene, is an ETS-family transcription factor with multiple roles in hematopoiesis. It is a lineage-specifying transcription factor that positively regulates many genes in the macrophage, granulocyte, dendritic-cell and B-cell lineages. Expressed at highest levels in monocytes/macrophages, at low or moderate levels in B cells, and transiently in early erythroid precursors, its action is also important or indispensable for sustained generation of all known hematopoietic precursors that have lymphoid developmental potentials (1–9). Thus, B, NK, and T cell development are all affected by defects in PU.1 activity, despite partial complementation by the related factor SpiB that is also activated in B-lineage precursors. Much is known about how PU.1 finds and binds to its sites in the DNA, typically (A/G)AGGAAGTG motifs [e.g., (10, 11)], and it is known to be able to bind either as a pioneer factor which displaces nucleosomes to open sites for other factors (12), or as a collaboration-dependent partner in binding complexes, either with activation-dependent factors like NF-κB or with lineage-defining partners like C/EBPα (or β) or IRF4/8 (13–15) [reviewed by (16–18)].

In myeloid, dendritic, and B lineage cells, PU.1 is a major contributor to the positive regulation of genes that establish lineage-specific identity (4, 17, 19). At the same time, PU.1 can work in an all-or-none gene network switch through mutual antagonism with GATA-1 (20–24), which has been much discussed as a possible mechanism for the irreversibility of erythro-myeloid lineage commitment [(25–29); but also see (30, 31)]. Nevertheless, the developmental scope of PU.1 activity is surprisingly broad, and one of its unexpected domains of action is in the early stages of T-cell development, in both the fetal and the postnatal mammalian thymus. To examine what it does in pro-T cells, this review focuses on recent data based on mouse T-cell development, mostly as it occurs in the postnatal thymus or from late fetal progenitors. The final section places these mechanisms in the context of the variants of T-cell development that characterize different ontogenic stages.

**Figure 1:**
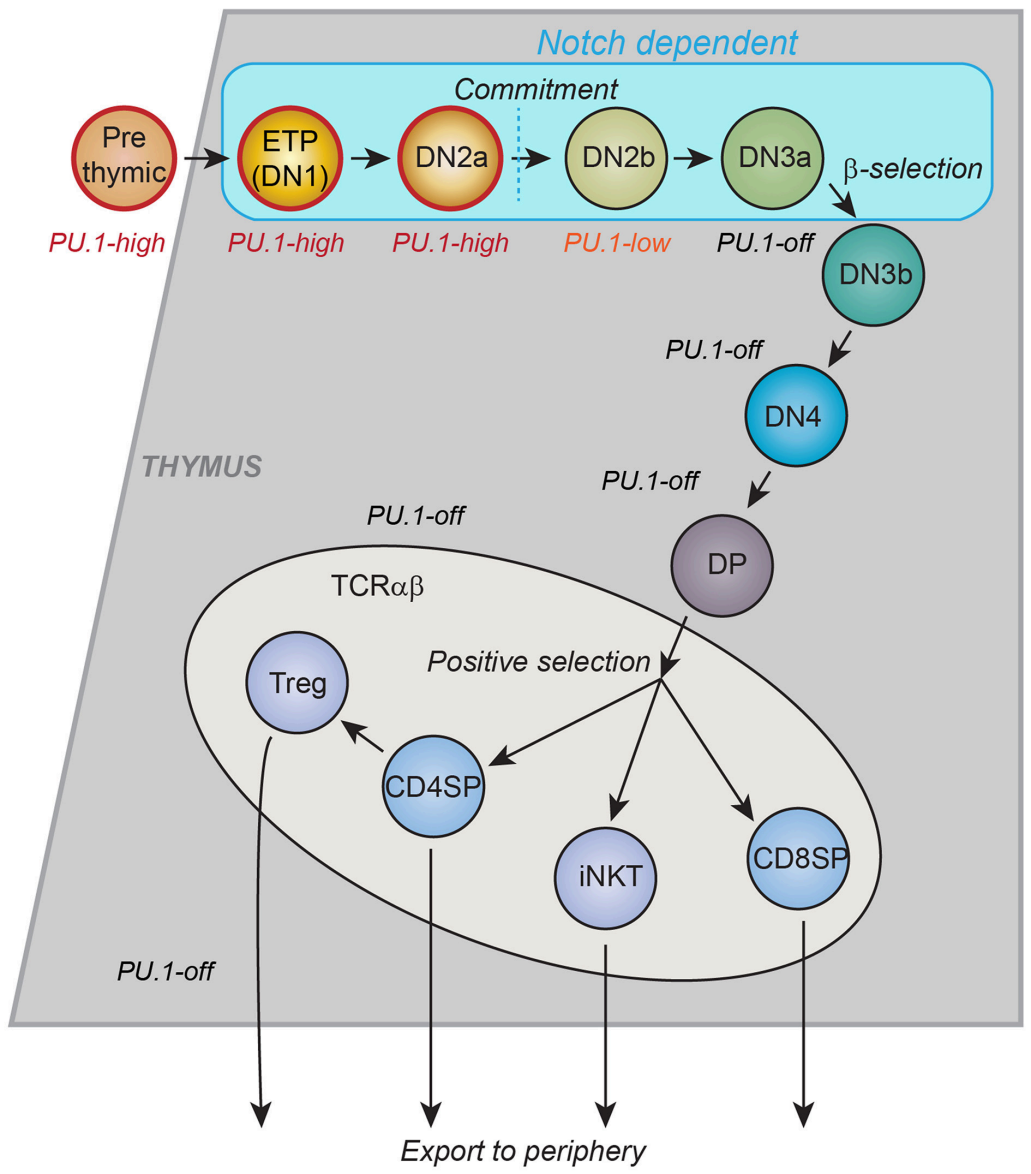
Schematic of T-cell development in the thymus. Major landmarks for T cell developmental stages are CD4 and CD8. CD4^−^ CD8^−^: “DN”; CD4^+^ CD8^+^: “DP,” CD4^+^ CD8^−^: “CD4SP”; CD4^−^ CD8^+^: “CD8SP.” All events described in this review occur within the DN stages, which are divided by other markers. ETP: Kit^++^ CD44^+^ CD25^−^; DN2a: Kit^++^ CD44^+^ CD25^+^; DN2b: Kit^+^ CD44^+^ CD25^+^; DN3a: Kit^−^ CD44^−^ CD25^+^ CD28^−^; DN3b: Kit^−^ CD44^−^ CD25^+^ CD28^+^; DN4: Kit^−^ CD44^−^ CD25^−^ CD28^+^. Stages up through DN3a do not depend on T-cell receptor gene rearrangement status and are called “Pro-T cells.” Many cell cycles occur between the ETP stage and commitment, more in post-natal T cell development and fewer in fetal T-cell development. The trends in PU.1 expression, the timing of intrinsic cell commitment to the T-cell lineage, and the stages that depend on Notch signaling from the thymic microenvironment are shown. Gray or blue regions depict thymic cortex. Lighter region depicts thymic medulla, where final maturation of developing T cells takes place. CD4SP: maturing T helper cells. CD8SP: maturing T cytotoxic cells. Treg: thymically derived regulatory T cells. iNKT: Natural Killer T cells with invariant T cell receptors [Schematic adapted from Rothenberg et al. (34)].

Most mature T cells do not express any detectable PU.1 protein or *Spi1* transcripts at all, and the T-cell developmental gene network sharply downregulates *Spi1* in precursors of αβ T cells before the expression of rearranged *Tcrb* genes, i.e., before any TCR-dependent steps of T cell development. However, the precursors that give rise to committed T cells express PU.1 at both RNA and protein levels for multiple cell divisions after these cells begin to differentiate in the thymus (32, 33). A summary of early T-cell developmental stages, is shown in Figure 1, with the approximate pattern of PU.1 expression marked. The downregulation of PU.1 occurs during the transition to commitment, between the DN2 (DN = double negative for CD4 and CD8, and Kit^+^ CD44^+^ CD25^+^) and DN3 (DN, and Kit^low^ CD44^low^ CD25^+^) stages. This expression timing relative to other developmentally regulated transcription factors is conserved between human and mouse (35, 36), and as in mouse (37), the downregulation of PU.1 is important to avoid malignancy in human T cells: a specifically aggressive class of human T-acute lymphoblastic leukemias results from translocations that promote abnormally sustained and elevated PU.1 expression (38). In the mouse, where lineage commitment has been studied in depth, there is good agreement between the cells' natural loss of access to the dendritic cell and granulocyte programs, on the one hand, and the timing of PU.1 downregulation, on the other hand (33, 39–42). This is part of a general downregulation of stem/progenitor associated regulatory genes (“phase 1 genes”) (34, 43) and a major reorganization of active chromatin and chromatin interactions, genome-wide, that occurs during this transition (44). One important question is what role PU.1 itself may have in controlling the onset of this transformation.

### PU.1 as an Obstacle to T-Cell Lineage Commitment

The particular interest in PU.1 itself emerged from the hypothesis that it could well be responsible for maintaining the “bridge” to myeloid and dendritic alternative fates before commitment, because of its known roles in many of these alternatives (and in B cells) but not in T cells (45). This hypothesis was supported by the finding that re-expression of PU.1 after T-cell commitment turns on myeloid genes and readily transforms later pro-T cells into dendritic cells, macrophages, or promyelocytic-like cells (46–50). There is a very close relationship between the cells that naturally express PU.1 in the thymus and those that readily exhibit myeloid or dendritic potential in a variety of permissive cell transfer models, *in vivo* or *in vitro*. Whereas ETP and DN2 cells can generate myeloid cells if removed from the thymus, pro-T cells that have differentiated past the stage of PU.1 expression in the thymus (i.e., from the DN2 stage to the DN3 stage or later) do not make myeloid cells under these conditions, and this difference between stages up to DN2 and stages from DN3 onward has been a highly consistent observation (33, 40, 41, 51–56). Why, then, do PU.1-expressing early T cell precursors within the thymus almost all go on to produce T cells, not myeloid cells, under normal *in vivo* conditions (57)? A potential explanation was provided by a key feature of the PU.1 effect: namely, that PU.1 actions are Notch-sensitive. Even artificially high-level PU.1 could only redirect the differentiation of the cells to myeloid or dendritic fates if Notch signaling were reduced (49, 50, 58). In primary fetal-derived pro-T cells and in a DN3-like cell line, the particular genes affected by a given, fixed level of PU.1 in the cells depended strongly on the strength of Notch signaling being induced in the cells at the time (58). Notch ligands are the most important of all the environmental signals that the thymus stroma provides to developing T cells, apparently across all vertebrates (59–61), and Notch activated target genes like *Hes1* are expressed throughout the pro-T cell phases (ETP to DN3), until T-cell receptor (TCR) gene rearrangement (62–64) [reviewed in (65)]. Thus, throughout the stages when PU.1 is expressed, the Notch signaling driven in the normal thymus environment could guarantee that PU.1 expression would confer only a potential for differentiation to alternative fates, which the cells would not actually follow unless the thymic environment were disrupted. The silencing of *Spi1* expression and permanent loss of PU.1 protein from the cells at a later stage of differentiation would then make their loss of myeloid potential unconditional.

The question raised by such results, however, was why PU.1 should continue to be expressed at all by cells once they entered the thymus. Population dynamic models imply that the stages when PU.1 is expressed occupy a minimum of 7–10 intrathymic cell divisions of pro-T cells (39, 66, 67). If PU.1 was evolutionarily selected to be expressed over such an extended period, it might be playing an important role in pro-T cells, and this could be despite or because of the Notch signaling conditions that were preventing it from diverting the cells to a non-T fate. The earliest stages of T cell development are not well understood, and it until recently it was not obvious what function could be important to the cells at this time *a priori*, other than proliferation. In the past 5 years, however, a detailed look at the molecular biology of PU.1 action on the genome in pro-T cells has revealed much about the ways that PU.1 works, the complex cell biology of the early precursor states, and previously under-appreciated principles of transcription factor systems operating in development.

## Effects of PU.1 Loss on T-cell Development: the Cellular View

### A Vital Role for PU.1 in Prethymic T-Cell Progenitors

Disruption of PU.1 has long been known to eliminate or greatly inhibit T-cell development, based on the dramatic phenotypes from the first lines of PU.1-knockout (*Spi1* knockout) mice with unconditional, germline mutations (68–71). The question has been how to interpret this severe effect, i.e., whether it is due to loss of a function within the T-cell program itself, or whether it simply reflects a loss of input cells to the pathway. One problem was originally the lethality of the hematopoietic phenotype (death either in late fetal development or immediately after birth), but even when conditional knockouts were developed (2, 72), this remained problematic. All the hematopoietic progenitors that generate either B or T cells appear to originate from PU.1-expressing, PU.1-dependent prethymic cells (2, 27); PU.1 is directly required to maintain the expression of the cytokine receptor Flt3 that is indispensable for progenitors with B and T cell potentials (73). Thus, in postnatal mice, although T cell development is much more severely affected by PU.1 deletion than neutrophil development (2), the effect could still be prethymic. In stark contrast, if *Spi1* is conditionally deleted in T-lineage cells only after the cells have passed the DN2 stage, there are very modest effects on T cells as a whole, apparently limited to selective reduction of IL-9-producing T-cells (74), and some loss of restraint on γδ T cells and T follicular helper cell activity (75, 76). Is PU.1 actually needed within the T-cell pathway for T-cell development at all, or is it simply needed to guarantee a supply of prethymic progenitors?

Addressing this question *in vivo* was handicapped by difficulties in the methods of inducing stage-specific *Spi1* deletion. The question about a transient role for PU.1, but one which might have strong effects on viability, makes it important to have high penetrance and high synchrony of deletion as well as fine developmental stage control, both of the deletion and through the analysis afterwards. The widely-used T-cell specific Cre expression constructs that might be appropriate for thymocyte analysis, pLck-Cre and CD4-Cre, actually begin to be expressed too late: pLck-Cre turns on just as PU.1 is turning off, and CD4-Cre is expressed even later, after the rearrangement of the first TCR genes. Constructs like Il7r-Cre or Rag1-Cre, which may have prethymic expession but are also expressed much more strongly during later pro-T cell stages, could make output cell phenotypes difficult to interpret because of uncertainty about when the deletion actually has become complete. Fortunately, pro-T-cell differentiation cultures on OP9-DL1 or OP9-DL4 stroma that constitively present Notch ligands (77, 78) are ideal for examining the stages relevant to PU.1 function, and a variety of efficient retroviral vectors can transduce the cells at these stages with high efficiency to introduce gain or loss of function agents. These systems have proven to be valuable tools not only for verifying the coarse-grained roles of PU.1 in pro-T cells, but also for investigation of their molecular mechanisms.

### PU.1 Promotes Proliferation While Slowing Differentiation of Pro-T Cells

PU.1 is indeed important within the T-cell program as well as before thymic entry, as shown by using *in vitro* differentiation to provide conditions where PU.1 could be removed acutely in a synchronized cohort of precursors and the fates of the cells could be monitored immediately afterwards. In these studies, floxed *Spi1* was disrupted in the input cells few days after T-lineage development had begun, using a Cre-encoding retroviral vector (79). The deletion of PU.1 reduced viable cell yield, but a co-transduced Bcl-xL transgene was added with Cre to prevent specific effects on development and proliferation from being masked by cell death. Similar results were obtained independently using Cas9 plus *Spi1*-specific guide RNA to delete PU.1, and supporting cell viability with a Bcl2 transgene (80). In both experimental setups, PU.1 disruption reduced T-cell precursor proliferation substantially as compared to controls. PU.1-deficient cells underwent fewer cycles per unit time than controls both in ETP stage and in DN2a/2b stages (79), suggesting that even once the cells have begun to express definitive T-lineage markers, they need PU.1 to sustain optimal proliferation. However, of the cells that were generated from PU.1-disrupted precursors, a substantially larger fraction progressed to DN3 stage than in control cells, over the same length of absolute developmental time, suggesting that they were liberated from a differentiation constraint (79, 80).

Thus, endogenous PU.1 does have a functional role within early T-cell development. It slows developmental progression of pro-T cells even as it supports their early proliferation. While this may seem paradoxical, it could fit well with a role to build the size of the pre-selection pool of T-cell precursors before they progress to commitment and then TCR gene rearrangement, so as to maximize TCR gene rearrangement diversity in the population as a whole before selection occurs (66, 81). The effect of PU.1 on proliferation is conditional and dose-dependent, however. While added PU.1 can enhance pro-T cell proliferation in response to cytokine cocktails containing high levels of Stem Cell Factor (Kit ligand) and Flt3 ligand or myeloid growth factors (38, 50), it strongly inhibits the proliferation of pro-T cells under conditions that do not reward the cells for lineage switching (49). Such dose dependent effects are common for transcription factors as for signaling molecules, in part because high concentrations of these factors bind to inappropriate genomic sites, leading to off-target effects. The target genes stimulated by PU.1 include both pro-proliferative and G1-prolonging cell cycle effectors, whereas some important proliferative genes are repressed when PU.1 levels are high (82, 83). Thus, both too much and too little PU.1 can have negative impacts on proliferation of the cells within a similar developmental time window.

The *in vitro* assays used to define these roles (discussed in more depth in the next section) are powerful because of the easy accessibility of the developing cells during differentiation and because of the ability to follow differentiation of a synchronized cohort of cells in absolute time. As described below, however, the genes most sensitively regulated by PU.1 in developing T cells suggest that this factor may be important to endow cells with additional functions as well, functions that may only contribute to their development specifically in the thymus *in vivo*.

## Defining the PU.1 Regulome in Early T-cell Precursors

### Cell Line and Primary-Cell Assay Systems for PU.1 Manipulation

**Figure 2:**
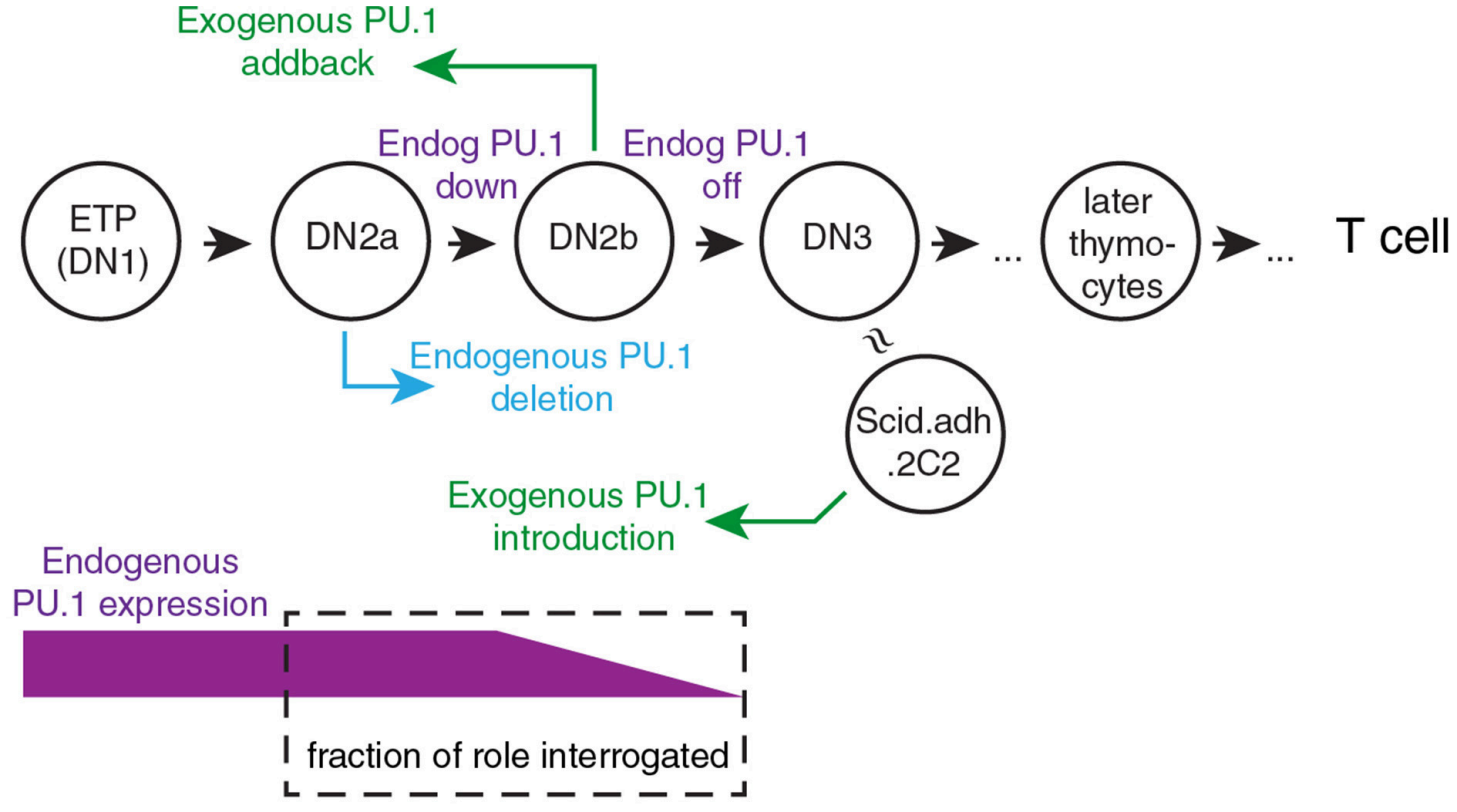
Framework for experimental perturbation studies to define functions of PU.1 in early thymic development. Stages of cells are as in Figure 1 (DN3: primarily DN3a). Exogenous PU.1 is added by retroviral transduction. Note the dependence of the PU.1 functions tested upon the timing of the experimental perturbation. Endogenous PU.1 can be deleted by Cas9 plus single-chain guide RNAs (sgRNA) against *Spi1*, or by introducing Cre into cells with floxed *Spi1* alleles. PU.1 can also be neutralized by adding a dominant negative construct. The DN2a-DN2b interval is accessible to experimental perturbation. The approximate developmental stage represented by the Scid.adh.2C2 cell line (see text) is also shown [Schematic adapted from Ungerbäck et al. (85)].

To explain the roles of PU.1 in T cell development, it is crucial to take into account its developmental expression pattern. Its high expression in early-stage pro-T cells followed by downregulation during commitment means that its direct effects have to be correlated with developmental stage. Thus, any inferred role must be validated by developmental stage-dependence of putative target gene expression patterns or of chromatin features that characterize its binding sites. To look more closely at how PU.1 actually regulates specific target genes, acute gain and loss of function experiments are needed. Despite some overlap in occupancy, PU.1 binding sites and PU.1 binding partners are not the same in early T-cell precursors as in myeloid cells or B lineage cells (13, 80, 84, 85), a similar situation to its early role in erythroblasts (86). Therefore, these assay systems need to be based on pro-T cells (Figure 2). Exogenous PU.1 can easily be introduced into developing murine T-cell precursors using retroviral vectors for gain of function studies (46–50, 58, 80, 85). For loss of function, retrovirally transduced Cre can induce acute deletion in pro-T cells from *Spi1*^*fl*/*fl*^ strain mice (79); and in Cas9-transgenic pro-T cells, retrovirally transduced guide RNAs (sg*Spi1*) can target rapid, biallelic disruption of the *Spi1* locus (80, 85). Figure 2 introduces the way the primary-cell and cell-line models can be manipulated to relate experimental gain-of-function and loss-of-function PU.1 experiments to the normal dynamics of endogenous PU.1 expression.

A very useful model cell line, Scid.adh.2C2, has made it possible to study PU.1 gain of function in a pro-T cell-like context (47). These cells are convenient because they are readily transfectable, retrovirally transducible, and fast-growing, so that cell numbers are not limiting and the developmental baseline is mostly static, all major advantages for genomic comparisons. These cells were a subclone derived from the Scid.adh cell line (87) and are similar to developmentally arrested versions of committed DN3 pro-T cells, lacking any expression of endogenous PU.1 (47). Despite being an immortal cell line, these cells are developmentally transformed by introduction of exogenous PU.1. They respond in an all-or-none way to forced expression of PU.1, coordinately upregulating myeloid- or dendritic-cell associated genes and downregulating T-cell genes in a discrete fraction of the cells that increases with increasing levels of PU.1 (47, 58), resembling responses of primary fetal or postnatal pro-T cells (46–50, 58, 80, 85). The switch-like nature of this response was an important early clue to the regulatory circuit interaction between PU.1 and the Notch signaling pathway (49, 58).

Useful and informative as it is, this system is limited as a way to study the roles of endogenous PU.1 *in vivo*. The sites occupied by exogenous PU.1 in Scid.adh.2C2 cells overlap highly with the sites occupied by endogenous PU.1 in normal pro-T cells, but the match is by no means complete (85). Even with PU.1 transduction, Scid.adh.2C2 cells do not restore the full chromatin accessibility landscape of ETP and DN2a stage pro-T cells, and despite detectable upregulation of a few other early pro-T cell genes (e.g., *Bcl11a* and *Lyl1*), the transduced cells as a whole reactivate little of the program that forms the normal context for endogenous PU.1 activity in pro-T cells (58, 79, 85). Therefore, PU.1 has to be manipulated acutely in dynamically differentiating primary cells.

To focus the introduction of PU.1 into cells at a particular developmental stage, it has proven to be very useful to exploit the powerful *in vitro* T-cell development systems based on co-culture of primary-cell precursors on OP9-DLL1 (aka OP9-DL1) or OP9-DLL4 (OP9-DL4) stroma with IL-7 and Flt3L (78, 88), or similar systems using other stromal cell lines to express the Notch ligands DLL1 or DLL4. Either fetal-liver-derived precursors or adult bone marrow-derived precursors develop efficiently along the T cell lineage in these systems with strong proliferation through the stages around commitment, allowing the stages to be separated both by flow cytometric phenotypes and by absolute times of differentiation. In these open systems, the cells can be harvested easily at any time point, transduced with vectors, treated with drugs, and/or sorted, and then shifted to the same or a different culture condition for further development. These systems have been indispensable for deeper analysis of the molecular mechanisms that PU.1 uses to regulate development of pro-T cells. However, two issues have to be taken into account in these analyses, both arising from features that amplify the developmental impact of PU.1. These are reviewed in the next sections.

### Developmental Challenges: Implications of a Gene Regulatory Network Switch

The ideal conceptual framework of PU.1 gain of function experiments is to start with pro-T cells that have recently turned off their endogenous PU.1 expression and to assess how their developmental state is affected by re-introducing PU.1 expression, comparing the impact of exogenous PU.1 with the pre-commitment gene expression pattern. Ideally in this scenario, restoring PU.1 after commitment should promote some aspect(s) of retrograde differentiation. Both Scid.adh.2C2 and normal DN2b/DN3 pro-T cells make strong responses to forced expression of PU.1, as noted above, and often the response includes downregulation of multiple later T-cell differentiation genes. Does this shed light on PU.1's natural role in earlier T-cell development, or is it simply an inhibitory artifact of overexpression? Clues that the gain-of-function phenotype is linked with a genuine role of PU.1 in earlier T-cell development come from PU.1's (re-)activation of a group of genes that are specifically associated with the early progenitor state, including *Bcl11a, Mef2c, Hhex*, and *Lmo2* (58, 79, 85). Some of these are also upregulated in human T-acute lymphoblastic leukemias with highly expressed PU.1 fusions, as well (38).

**Figure 3:**
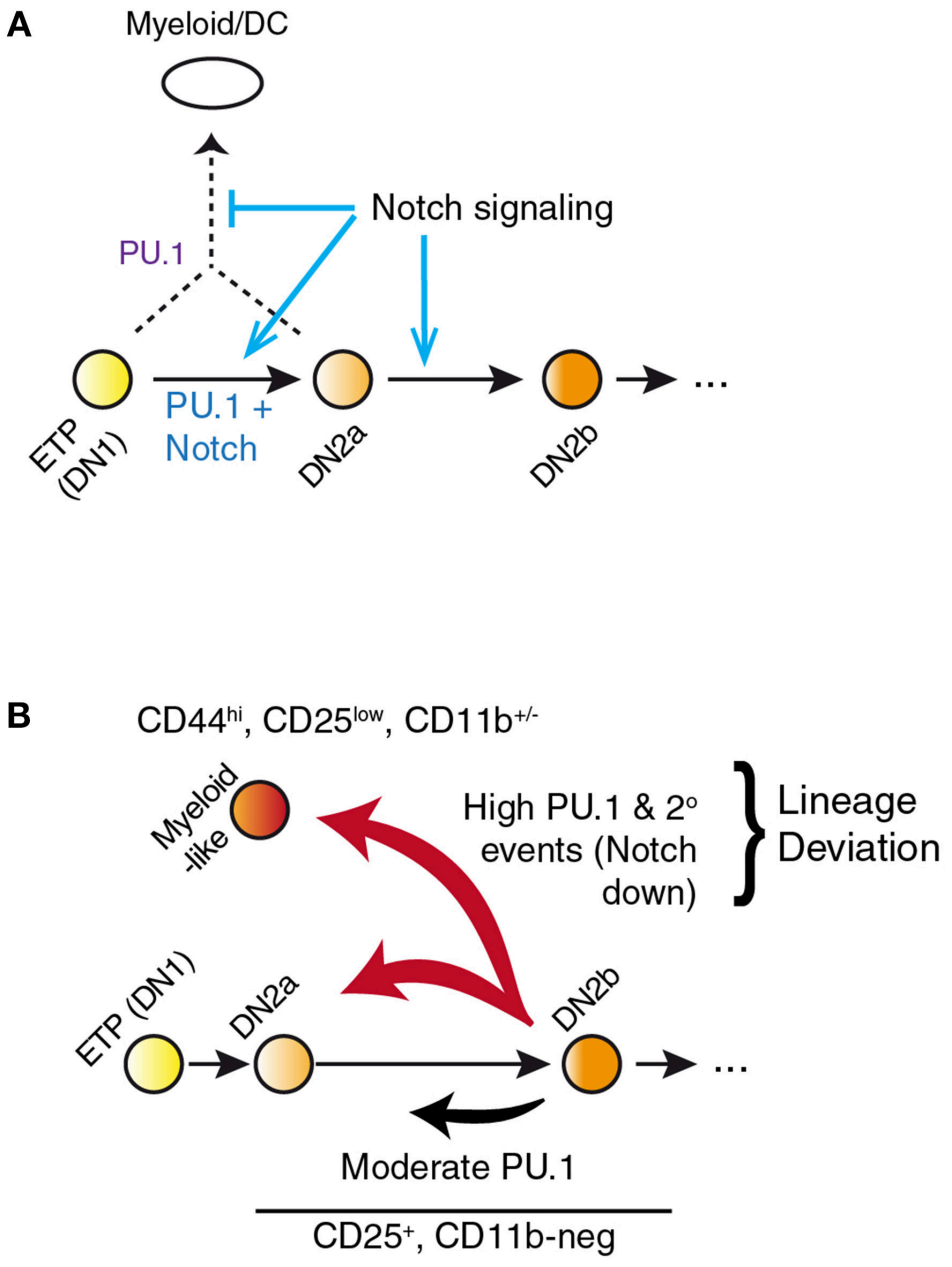
PU.1 and Notch collectively determine T vs. myeloid lineage fates. **(A)** Notch signaling within the thymus normally constrains PU.1 effects to support early pro-T cell development while blocking alternative fates that PU.1 would otherwise promote. Notch signaling itself does not repress PU.1 expression; however, other transcription factors induced by Notch signaling eventually silence expression of *Spi1* during the DN2b stage. **(B)** Separation of the effects of PU.1 within the T-cell pathway from effects of PU.1 to promote lineage deviation, in PU.1 gain of function experiments. A fraction of cells expressing high levels of PU.1 shift to a myeloid-like state that can be phenotypically distinguished from cells remaining within the T-cell state. This distinction is necessary to relate gain-of-function effects of PU.1 in pro-T cells to effects of loss of endogenous PU.1 in perturbation experiments. Lineage deviation is associated with a broad loss of Notch signal response in the cells, suggesting that the constraint mechanism shown in A has been overwhelmed in these cells. Biochemical mechanisms of these effects remain to be fully defined. Schematic in **A**, adapted from (58); in **B**, adapted from (85).

However, the power of the response raises caveats about interpretation because of an important systemic feature of the PU.1 role in development. In primary pro-T cells and Scid.adh.2C2 cells, highly overexpressed PU.1 appears to inhibit Notch signaling, as measured by downregulation of Notch target genes and even *Notch1* itself. Whether cause or effect, this collapses the balance between Notch signaling and PU.1 activity that is fundamental to channel the natural role of PU.1 in early pro-T cells (see above) (49, 58). The most pronounced effects of PU.1 are thus a nonlinear response to PU.1 dosage mediated through a gene network switch (Figure 3), and this gene network switch underlies the stochastic, switch-like behavior of individual pro-T or Scid.adh.2C2 cells when forced to express high-level PU.1 (47, 58). Importantly, the combination of PU.1 with Notch signaling desensitization pushes the cells out of the T-cell program completely, rather than simply reversing their progression through the T-cell program. Instead of re-acquiring aspects of a progenitor-like state, the cells appear to trans-differentiate to a dendritic-cell or macrophage developmental program (48, 50, 58).

**Figure 4:**
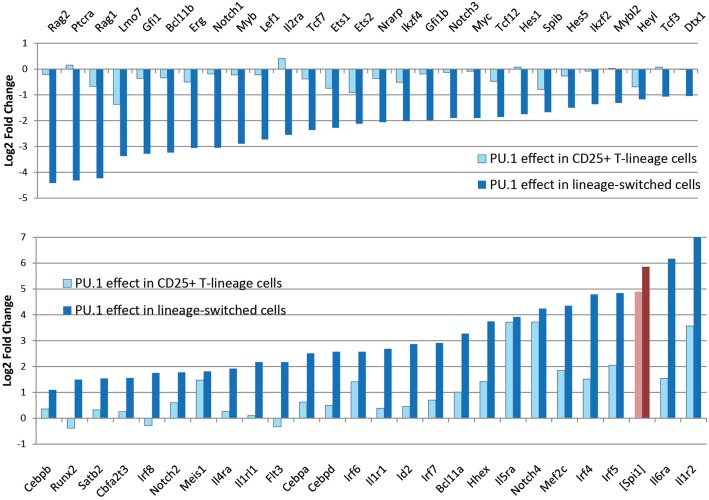
Profound changes in regulatory gene expression distinguish PU.1-induced lineage deviation from PU.1 effects within the T-cell pathway. Charts show changes in expression of the indicated genes (log_2_ Fold Change relative to controls) induced by introduction of PU.1 into post-commitment pro-T cells (DN2b-DN3). Panels compare effects on T-lineage regulatory genes **(top)** and non-T regulatory and signaling genes **(bottom)** between cells remaining within the T-lineage pathway (light blue bars) and cells undergoing lineage deviation (dark blue bars). Light, dark red bars show corresponding measured levels of exogenous PU.1 in these samples, as log_2_ fold changes over controls, which have downregulated most of their endogenous PU.1 expression at this stage. Results are from Ungerbäck et al. (85).

In newly-committed primary pro-T cells forced to express PU.1, the cells crossing this developmental boundary are seen to downregulate the Notch-dependent DN2/DN3 stage marker, CD25 (*Il2ra*), and often upregulate the myeloid-associated marker CD11b (Mac1; *Itgam*). The gene expression profiles of cells losing CD25 and upregulating CD11b are radically transformed from the state of newly-committed pro-T cells within 2 days after transduction, with widespread repression of T-lineage-affiliated transcription factor genes and Notch target genes as well as upregulation of multiple *Cebp* and *Irf* family transcription factor genes (Figure 4) (85). This response is quite different in gene expression pattern from retrograde differentiation to an ETP- or DN2a-like phenotype. In contrast, cells remaining within the T-cell pathway, continuing to express CD25 and remaining negative for CD11b, show relatively modest and selective changes in gene expression driven by upregulated PU.1, with minimal loss of T-cell regulatory gene expression (79, 85). Details of these transcriptome effects are discussed in a later section, but the point here is that they include qualitative as well as quantitative differences in the gene expression responses. The differences in average *Spi1* overexpression levels between cells making these two responses are only on the order of ~2–3 fold (pink, dark red bars in Figure 4), so it is very likely that the additional changes in other regulatory genes contribute strongly to this global shift. Thus, the effect of PU.1 expression *per se* may be part of the normal T-cell program, but under high-level expression conditions it combines with additional, conditionally induced mechanisms to produce a much broader spectrum of developmental effects that may not only be direct responses to PU.1 itself.

### Kinetic Challenges: Protein Half-Lives vs. Developmental Progression

Loss of function approaches are indispensable to confirm the roles of endogenous PU.1, especially in view of the potential for indirect effects in gain of function experiments, just described. Here, the challenge has been to find a way to remove or neutralize the endogenous factor quickly enough to see effects robustly, while keeping the controls and the experimental samples at comparable developmental stages. One problem is that the long half-life of PU.1 protein (82) can mask some loss effects at time points <2 days after deletion, while development of the pro-T cells can proceed to new stages if time windows are extended further. There are thus several problems with generating high-quality samples for analysis of transcriptome changes caused by PU.1 loss of function. Cre-dependent deletion of a loxP-flanked *Spi1* allele (*Spi1*^*fl*.*fl*^) is asynchronous, and in an early T-cell population with mixed degrees of *Spi1* deletion, cells with inadequate PU.1 levels appear to be at a selective disadvantage, even *in vitro*. Ironically, because PU.1 protein can persist longer than a cell cycle (82), the very slowdown of cell division caused by deletion of *Spi1* (see above) can also interfere with the dilution needed to complete the clearance of the PU.1 protein. As a result of the enrichment of cells with undeleted alleles, and this persistence of pre-existing PU.1 protein even from the cells that have successfully deleted its coding gene, the effects on target gene regulation appear very weak at timepoints up to 2 days after PU.1 deletion, despite the fact that the reduced cell yields from the knockout cells show that PU.1 is biologically important (79). If timepoints are taken too long after deletion, the controls progress to the point when endogenous PU.1 is downregulated, so that any truly PU.1-dependent targets are expressed weakly in the controls, and comparisons with the knockout samples again lose statistical power. A very intriguing new prospect for fast antagonism of PU.1 activity is the discovery of small-molecule inhibitors, some of which are highly potent and specific at blocking PU.1 action in leukemia cells; however, these have not yet been tested for effects on normal T-cell development (89).

**Figure 5:**
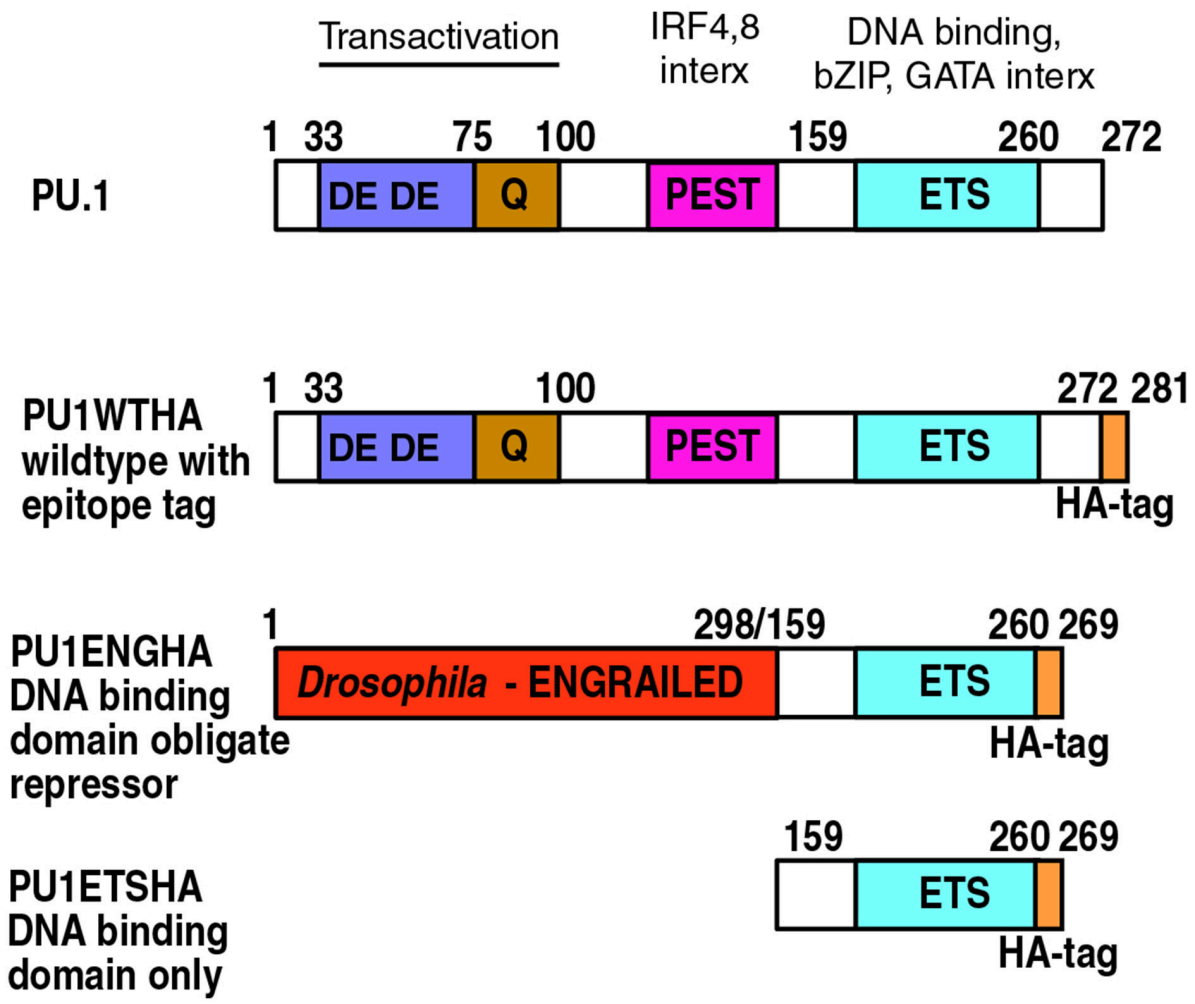
PU.1 structure and derivatives of PU.1 used for functional analysis. Domain boundaries within the amino acid sequence of murine PU.1 are depicted with their associated functions indicated at the top (4, 18, 90, 91). Epitope-tagged wildtype PU.1 (PU1WTHA) and two epitope-tagged, modified constructs are shown (PU1ENGHA, PU1ETSHA); these are used to interrupt endogenous PU.1 activity (79, 82). PU1ENGHA and PU1ETSHA have a full DNA binding domain and efficiently enter open chromatin, but are deficient in entering closed chromatin (85). DE: Acidic residue-rich transactivation domain. Q: Glutamine-rich transactivation domain. PEST: Proline, Glutamate, Serine and Threonine-rich domain, site of IRF4 and IRF8 interaction (interx). Note that in PU.1 this “PEST” domain does not make the protein unstable. ETS: E-twenty-six proto-oncogene homology domain, the DNA binding domain of PU.1. This is also the region that interacts with basic leucine zipper (bZIP) factors such as Jun and C/EBP factors, and GATA family factors.

A relatively fast way to neutralize PU.1 protein activity directly has been to transduce the cells with a “dominant negative” obligate repressor derivative of PU.1, a fusion protein of the PU.1 DNA binding domain with the repression domain of *Drosophila melanogaster* Engrailed (PU.1-ENG), to compete for binding against endogenous PU.1 (79) (comparison with wildtype PU.1 shown in Figure 5). The obligate repressor should affect PU.1 positive regulation targets in the opposite direction from wildtype PU.1, and in theory should affect PU.1 negative regulation targets in the same direction, an “algebraic sign” distinction that could be used in principle to dissect indirect effects as well (79). This construct has been useful to reveal quick impacts on expression of positively regulated PU.1 target genes, many of which have been confirmed later by other approaches (85). For example, whereas PU.1 itself can upregulate progenitor-associated genes *Bcl11a, Lmo2, Mef2c*, and *Hhex* above their normal levels in DN2a and DN2b primary cells, PU.1-ENG can downregulate them (79). However, PU.1-ENG also has some spurious effects and cannot access closed chromatin sites as well as full-length PU.1 (79, 85).

Cas9-dependent acute deletion of the *Spi1* locus can be fast and highly efficient due to the availability of Cas9-transgenic mice (92) and vectors that can be used for high-level, synchronous expression of guide RNAs. However, deletion and clearance of PU.1 protein in this system still require analysis >2 days after introduction of the guide RNAs (80, 85), and the continuing developmental progression of both knockout samples and controls needs to be taken into account in interpreting the results. The strongest evidence for specific physiological PU.1 effects therefore comes from the consensus results from two or more of these perturbation systems. The highest confidence list of potential PU.1 target genes in pro-T cells could be defined as genes that responded reciprocally to gain and loss of PU.1 function within the same DN2a-DN2b developmental interval, and these genes are listed in Table 1. While this list under-represents some PU.1 targets that are only expressed in ETP stage, rigorous definition of the genes that are directly regulated by PU.1 in pro-T cells has made it possible to investigate the range of mechanisms used by the PU.1 protein to exert these transcriptional effects.

**Table 1:** High confidence targets of PU.1 regulation in pro-T cells.

**Consensus PU.1-repressed target genes**	**Consensus PU.1-activated target genes**
2900079G21Rik	3110043O21Rik
Ablim1	5430427O19Rik
Adamts9	9930111J21Rik1
Adora2a	9930111J21Rik2
Arsi	Abcb1b
Bcl2	Acer3
Ccnd3	Actn1
Cd247	Actr2
Cd28	Acy1
Cdc25b	Adam11
Cecr5	Adam15
Clec2i	Adap1
Clic5	Adgre1
Csrnp1	Adrbk2
Cx3cr1	Alcam
Cxcr5	Alox5ap
Cxxc5	Anks3
Dgka	Antxr2
E2f2	Ap1s3
Eng	Apbb1ip
Epcam	Aqp9
Fam160a2	Arhgap6
Gbp10	Arhgef40
Gbp11	Atp13a2
Gbp4	Atp6v0a1
Gbp6	Avpi1
Gimap1	B3gnt7
Gimap4	BC035044
Gimap6	Bcat2
Gimap8	Bex6
Gimap9	Bloc1s2
Hdac4	Bri3bp
Hid1	Btk
Hsd11b1	Cacnb2
Il12rb1	Ccdc180
Ilvbl	Ccl9
Irak3	Ccnd1
Itk	Cd180
Jph2	Cd300a
Lmo7	Cd300lf
Lztfl1	Cd33
Mbp	Cd34
Mir1903	Cd44
Neil1	Cdh1
Nipal1	Clec10a
Pik3ip1	Col9a3
Pitpnc1	Coro2a
Pitpnm2	Cotl1
Ppm1h	Creg1
Prf1	Crtac1
Ptprf	Csf2rb2
Rab27a	Csgalnact2
Rdh10	Ctbp2
Repin1	Cyp4f18
Selplg	Dnase2a
Sh2d5	Dock5
Sh3bp5	Dstyk
Slc11a2	Ebi3
Slc12a7	Entpd1
Slc27a1	Erlin1
Sox13	Erp29
Spata6	Fam101b
Spib	Fam217b
Spo11	Fam49a
Spry1	Fcgr2b
Ssbp2	Ffar2
Sstr2	Fgd2
Tas1r1	Fgr
Tecpr1	Fh1
Tlr12	Fig4
Tmc8	Flnb
Tnfsf11	Gapt
Tox2	Gfod1
Traf3ip2	Gm16712
Trat1	Gm16897
Trp53inp1	Gm2a
Tspan13	Gng10
Tspan32	Gng2
Utrn	Gns
Wnt5b	Gpx1
	Gucy1a3
	Gusb
	Haao
	Hbb-b1
	Hbb-b2
	Hbb-bs
	Hbb-bt
	Hck
	Hfe
	Hpse
	Hsd17b6
	Idh2
	Il12rb2
	Il13ra1
	Il1r2
	Il5ra
	Inpp5j
	Irf5
	Irf6
	Itgad
	Itgam
	Itgax
	Jak2
	Kcnk12
	Kcnk6
	Khdc1a
	Khdc1c
	Klhl18
	Kmo
	Krt80
	Lair1
	Lmo1
	Lpcat2
	Lrba
	Lrrc25
	Lrrc75a
	Lst1
	Ltb4r1
	Ltbr
	Lyn
	March1
	Matk
	Mb21d1
	Mef2c
	Megf8
	Met
	Mfsd12
	Myo1f
	Naaa
	Nccrp1
	Ncf1
	Ncf2
	Ndst1
	Ndufb8
	Nedd9
	Neurl3
	Nfam1
	Nlrc4
	Nlrp10
	Nlrp1b
	Nod2
	Npl
	Nuak2
	Oas1a
	Oas2
	Ogfrl1
	P2ry13
	P2ry14
	Padi2
	Pak1
	Pdxk
	Phactr2
	Pik3ap1
	Pik3r6
	Piwil2
	Pla2g4a
	Plac8
	Pld4
	Plek
	Plxnd1
	Pmvk
	Pqlc1
	Prex1
	Prkcd
	Prtn3
	Ptpn6
	Ptpre
	Rab31
	Ralb
	Rcn3
	Relt
	Rgs18
	Rnf149
	Rogdi
	Rufy1
	Samhd1
	Sema3c
	Serpina3g
	Sh2b2
	Sh3pxd2a
	Siglece
	Siglecf
	Siglecg
	Skap2
	Sla
	Slc16a7
	Slc35d3
	Slc8a1
	Snx10
	Sorl1
	Spi1
	Stx7
	Susd3
	Svip
	Syk
	Tbc1d24
	Tbxas1
	Tdrd7
	Tgm1
	Themis2
	Tlr9
	Tmc5
	Tmem51
	Tmprss3
	Tnni2
	Tor3a
	Trim55
	Trmt2a
	Trpm2
	Tyrobp
	Ufsp2
	Unc93b1
	Vamp8
	Vps18
	Wdfy4
	Ywhag
	Zc3h12d
	Zfp385a
	Zfp52

*This table lists high-confidence PU.1-regulated genes which show reciprocal responses in the loss of function and gain of function perturbations of PU.1. The lists give the three-way intersection of genes affected in loss of function, and (reciprocally) in gain of function within cells remaining CD25^+^, and in gain of function within cells becoming CD25^−^ CD44^+^. Consensus PU.1-repressed target genes: genes with expression that goes down in PU.1-transduced DN2b with or without lineage diversion (CD25^+^ or CD44^+^ cells), and also increases in normal DN2a cells after deletion of endogenous PU1. Consensus PU.1-activated target genes: genes with expression that increases in PU.1-transduced DN2b with or without lineage diversion, and also goes down in normal DN2a cells after deletion of endogenous PU.1. Data compiled from Ungerbäck et al. (85)*.

## PU.1 Action on the Genome via Direct Binding

### PU.1 Protein Is Stable and Active Across the Genome in Early T Cells

Most of the initial hypotheses about PU.1's role in T-cell precursors were based on *Spi1* RNA expression patterns and on forced expression of exogenous PU.1 to supra-physiological levels (46, 48–50). With the advent of ChIP-seq data, though, it was confirmed that endogenous, naturally expressed PU.1 is indeed a prominent actor across the genome in T-cell precursors before commitment. PU.1 was found binding to >30,000 genomic sites in these cells at the earliest stages (84), and intracellular protein staining confirmed that some PU.1 expression is still detectable at later stages, in the same individual cells that go through T-cell commitment (marked by activation of the *Bcl11b* gene) (33, 93). In addition, although the RNA transcript levels are modest in absolute terms, the impact of PU.1 on the cells can be magnified by the high stability of PU.1 protein (82). Although PU.1 occupancy of genomic sites declines as development proceeds, PU.1 occupancy is still detectable through T-lineage commitment at ~5,000 sites before disappearing (84).

### PU.1 Binding Site Characteristics

The sites where PU.1 binds are enriched for open chromatin as defined by DNase accessibility or ATAC-seq [assay of transposase-accessible chromatin (94)], and circumstantial evidence suggests that PU.1 is a major factor at those sites that change activity during commitment. PU.1 recognition motifs are the most highly enriched of all defined motifs at sites that start out highly accessible in early pro-T cell stages, when PU.1 is present, and lose accessibility during commitment, i.e., as PU.1 levels decline (44, 85, 95). PU.1 itself is functionally important for the open status of these chromatin sites in the early stages, for many of these sites in fact do not remain as open if the PU.1 is removed acutely from primary pro-T cells by Cas9-mediated deletion (85). This is consistent with PU.1's activity as a site-specific chromatin opening factor in B cells (96, 97), with the ability of PU.1 to eject nucleosomes from sites where it binds in macrophage lines (12), and with its ability to cause rapid increases in ATAC accessibility at the sites it occupies when introduced into Scid.adh.2C2 cells (85). While PU.1 binds at both promoters and non-promoter sites, the evidence from both gain and loss of function studies shows that PU.1 is most associated with chromatin accessibility when it is binding at non-promoter sequences, within introns of genes or in intergenic regions. As described in detail below, such sites, where PU.1 itself is important to maintain chromatin accessibility, are the ones most often linked to genes that are positively regulated in their expression by PU.1 (85). Thus, PU.1 action to keep sites open in chromatin may be an important way that it promotes transcriptional activation in pro-T cells.

Because PU.1 mediates different effects in the rather different regulatory contexts of B, dendritic, myeloid, erythroid progenitors and pro-T cells, an important question is how much of PU.1's binding choice hierarchy is dependent on the prior epigenetic history of cells. PU.1 cannot enter all genomic sites. Notably, PU.1 appears to be excluded from genomic regions that are packaged in Polycomb Repressive Complex 2-modified chromatin, as marked by trimethylation of Histone H3 Lysine 27 (H3K27me3) (84). However, approximately half of the PU.1 occupancy sites in early pro-T cells appear to be relatively “inaccessible” in chromatin by the criterion of ATAC-seq at the stages when PU.1 is seen to be binding there, showing that PU.1 binding can occur without opening the chromatin. These sites in closed chromatin have particularly high-quality matches to the consensus PU.1 binding position weight matrix (85), which several lines of evidence show to be a good indicator of PU.1 binding affinity (11, 85). This suggests that closed chromatin may be less permissive to PU.1 binding than open chromatin, so that only high-affinity site recognition allows binding in closed regions. However, this stringent specificity criterion also shows that these are not “off-target” sites: the tradeoff between site accessibility and the affinity of binding needed for occupancy indicates that PU.1 itself is identifying these sites in closed chromatin to establish its occupancy. By the ability to enter closed chromatin at its own high-affinity sites, and by its functional role in controlling chromatin accessibility at other sites, PU.1 meets the criteria for “pioneer” factor activity in early pro-T cells (98), and the scope of its binding suggests a broad role in genomic architecture of these cells.

**Figure 6:**
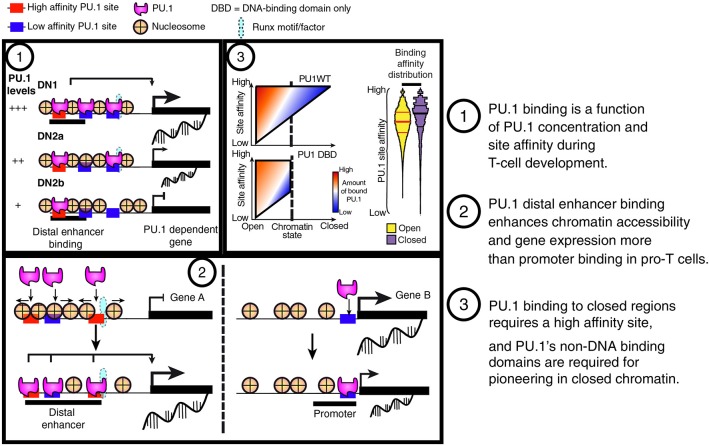
Summary of PU.1 binding features in pro-T cells. (1) PU.1 binding is preferentially retained at high-affinity sites as pro-T cells progressively reduce their PU.1 levels. This feature indicates that PU.1 primarily uses mass action (concentration × affinity) to determine its genomic site choices in these cells. (2) PU.1 works as a positive regulator in pro-T cells primarily by binding and controlling accessibility of sites distal to the transcriptional start sites, not at promoters. (3) The binding profiles of full-length PU.1 (PU1WT) show a tradeoff between binding site affinity and binding site accessibility in chromatin; however, constructs with the PU.1 DNA binding domain but lacking the transactivation domains (PU1 DBD) are poor at engaging sites in closed chromatin no matter how high their potential affinities.

Still to be determined are the rules determining when PU.1 binding to closed chromatin results in opening of the closed site. A priori, one could imagine that PU.1 establishes occupancy using its DNA-binding domain and then uses its protein-interaction domains to recruit chromatin modifying complexes (Figure 5, diagram of structures). Many of the known protein-protein interactions between PU.1 and other transcription factors on the DNA are also mediated through parts of the DNA binding domain, consistent with a compartmentalized role of this domain of the protein for binding site choice [reviewed in (18)]. However, PU.1 also contains non-DNA binding domains, acidic and glutamine-rich “transactivation” domains and an IRF4/8 binding domain, that are also clearly implicated in PU.1 function, as selective deletions of these domains greatly reduce PU.1 developmental impacts (24, 47, 99). Recent evidence has pointed to another role of the non-DNA binding domains of PU.1, a function needed for PU.1 to enter closed chromatin. Genome-wide, exogenously introduced full-length PU.1 and the isolated PU.1 binding domain establish quantitatively similar patterns of occupancy at open sites and especially at open promoters all across the genome. However, they show a marked difference in binding between open and closed chromatin, especially at non-promoter sites. Full-length PU.1 binds closed sites nearly as well as open sites, whereas the isolated PU.1 DNA binding domain binds open sites as well as full-length but selectively fails to bind at closed sites (85) (Figure 6). This suggests that an additional process beyond simple DNA sequence recognition is required to establish PU.1 occupancy in closed chromatin, even without an overt change in chromatin accessibility as measured by ATAC-seq. A similar mode of action has already been described for a separate, non-DNA binding domain to enable EBF1 entry into closed chromatin (100). It will be interesting to see if this is a general feature of pioneer transcription factors.

## PU.1 Action via Cofactor Recruitment

### Direct Gene Regulation by PU.1: Activation by Distal Enhancer Engagement and Opening

The major problem with connecting PU.1 binding to PU.1 regulatory function is that PU.1 binds to too many genomic sites in ETP and DN2a pro-T cells (84). It shows high fidelity in terms of sequence recognition, but its binding is not confined to functionally responsive target genes. It is found at a large fraction of open, accessible chromatin elements during the stages when it is expressed, and often bound at promoters as well as distal elements. However, only a minority of the genes linked to its binding sites change expression at all across the developmental interval when PU.1 goes from full expression to silence (84). Much of PU.1 binding in pro-T cells thus appears to be either functionally redundant or opportunistic. Identifying PU.1's functional mechanisms of target gene regulation has required a way to link an experimentally inducible *change* in PU.1 binding at a given site with the rapid, measurable *change* in expression of the target gene linked to that site. This is considerably easier to do in a gain of function format than in loss of function, as an epitope-tagged exogenous PU.1 construct can be introduced with fast kinetics and its newly established binding tested for association with local gene expression responses (85). Note that the gain-of-function experimental design makes it necessary to use another criterion to screen out genes that are only indirectly affected, as described above. For this reason, in our recent study (85) only cells that remained CD25^+^ CD11b^−^ (see above) were used for ChIP-seq analysis of exogenous PU.1 binding.

The results showed that PU.1 exerts its main functional regulatory impacts in pro-T cells via non-promoter sites, and especially via sites that are normally developmentally changing in chromatin accessibility (85) (Figure 6). In the aggregate, most of the responses of genes linked directly to PU.1 binding sites were positive; direct repression targets were much rarer. Genes responding to the addition of exogenous PU.1 usually had the exogenous PU.1 binding to distal (intronic or neighboring intergenic) sites, whereas genes that had PU.1 binding only to their promoter regions usually did not change expression at all. In the “blank slate” background of the Scid.adh.2C2 cell line, exogenous PU.1 binding opened chromatin at its non-promoter sites within 2 h, increasing the “activating” H3K27Ac marks at these sites a few hours later, and the linked genes were predominantly upregulated within 8–24 h. The genomic sites that were most highly associated with these responses in primary pro-T cells were developmentally dynamic in chromatin accessibility: normally open in early stages of T-cell development (endogenous PU.1-expressing) but closed once the cells went through commitment (endogenous PU.1-low or negative). Thus, the sites in pro-T cells with the strongest sensitivity to exogenous PU.1 for transcriptional impact were also sites where endogenous PU.1 might be important for maintaining chromatin accessibility.

### The Problem of Pro-T Cell Gene Repression by PU.1

The impact of PU.1 on pro-T cell gene expression overall is at odds with the biochemical and genomic evidence for its mode of action in one respect: PU.1 introduced into primary pro-T cells or Scid.adh.2C2 cells causes downregulation of many T-cell genes, especially those associated with Notch signaling and TCR gene rearrangement after commitment. This response is fast, reducing existing transcript pools for many repressed genes even before most positively regulated PU.1 target genes are seen to be turned on (80). However, the local impact of PU.1 binding is strongly biased toward activation of genes linked to the binding sites. While much of the data showing T-lineage affiliated gene downregulation comes from forced PU.1 re-expression or overexpression experiments, and might therefore be a high-dose artifact, it is important to note that the developmental speed-up observed in primary pro-T cells when PU.1 is knocked out also points to a normal PU.1 role as a brake on developmental progression (79). Thus, to account for PU.1's overall role, some explanation for the repressive outcomes is essential.

There is a long history of research on PU.1 as a repressor of genes associated with non-myeloid pathways, especially in the context of PU.1—GATA-1 antagonism in hematopoiesis [reviewed in (4, 7, 25)]. At high levels, PU.1 has been found to block DNA binding by GATA-1 (23), while at lower levels it is reported to antagonize GATA-1-mediated transactivation by forming complexes with it that recruit Rb through the PU.1 acidic transactivation domain (101). It is also reported to act as a repressor by direct recruitment of Dnmt3b (102). In most of these cases, PU.1 is observed to bind directly to the regulatory DNA of its repression targets (86, 103). However, in the pro-T cells, the genes that are repressed when exogenous PU.1 is introduced are not necessarily linked to the sites that the exogenous PU.1 actually binds. In fact, results with the PU.1-ENG obligate repressor construct implied that some kind of indirect effect must be involved: while the obligate repressor downregulated genes that are positive regulatory targets of wildtype PU.1, it actually upregulated many genes that wildtype PU.1 represses, completely inconsistent with a direct repression mechanism (79).

To date, three mechanisms appear to be involved. First, as noted above, high-dose PU.1 can inhibit expression of multiple Notch target genes and *Notch1* itself (58, 85). It is possible that the fast downregulation of Notch response genes, including *Hes1, Nrarp, Dtx1, Lef1*, and *Il2ra*, by overexpressed PU.1 is due to the loss of positive Notch signaling input rather than to a gene-specific mechanism. This Notch-inhibitory mechanism is not operating in cells that remain within the T-cell pathway, but it becomes prominent in cells that PU.1 causes to transdifferentiate, and would be expected to affect all T-cell genes that use Notch signaling as an obligate positive input, whether or not PU.1 binds them directly. A related scenario in which PU.1 could interfere with a T-lineage specific positive regulatory input might be through repression of GATA-3 by PU.1, by analogy with the cross-inhibition of PU.1 and GATA-1. However, in pro T cells, both PU.1 and GATA-3 are active together and both functionally important throughout the ETP to DN2a stages (79, 104), and there is more evidence for GATA-3 repression of PU.1 than for PU.1 repression of GATA-3 (58, 79, 104, 105). However, GATA-3 function also may become a casualty of PU.1 action when Notch signaling is inhibited (58).

Second, in pro-T cells forced to express PU.1, those that make the lineage jump (i.e. lose CD25, gain CD11b) not only silence *Notch1* but also activate myeloid regulatory genes (85). They also begin to express multiple transcription factors of the Egr and IRF families, and in the case of primary cells, they also upregulate C/EBP family factors. These factors probably contribute independently to the repression of pro-T cell genes. Egr2, for example, can collaborate with PU.1 in positive regulation when co-bound with it (106), but has also been implicated as a PU.1-stimulated repressor of the *mir17*~*92* complex (107). Although not required for *Notch1* repression in Scid.adh.2C2 cells (58), when activated in primary cells, C/EBPα itself can also repress *Notch1* (50). PU.1 does not strongly upregulate transcription factors annotated as repressors, however, in cells remaining within the T-cell program (see below). Therefore, this indirect repressive activity, too, would only be deployed under conditions of lineage shift.

The third mechanism that could play a role in repression within the T-cell program comes from PU.1's own ability to recruit other transcription factors to collaborate with it at PU.1 binding sites. This is a hallmark of pioneering activity in developmental gene regulation (98, 108), but in this case it exposes a particularly intricate post-transcriptional relationship between PU.1 and the factors required for progression of the T-cell program.

### System Consequences of Cofactor Recruitment: Repression by Theft

**Figure 7:**
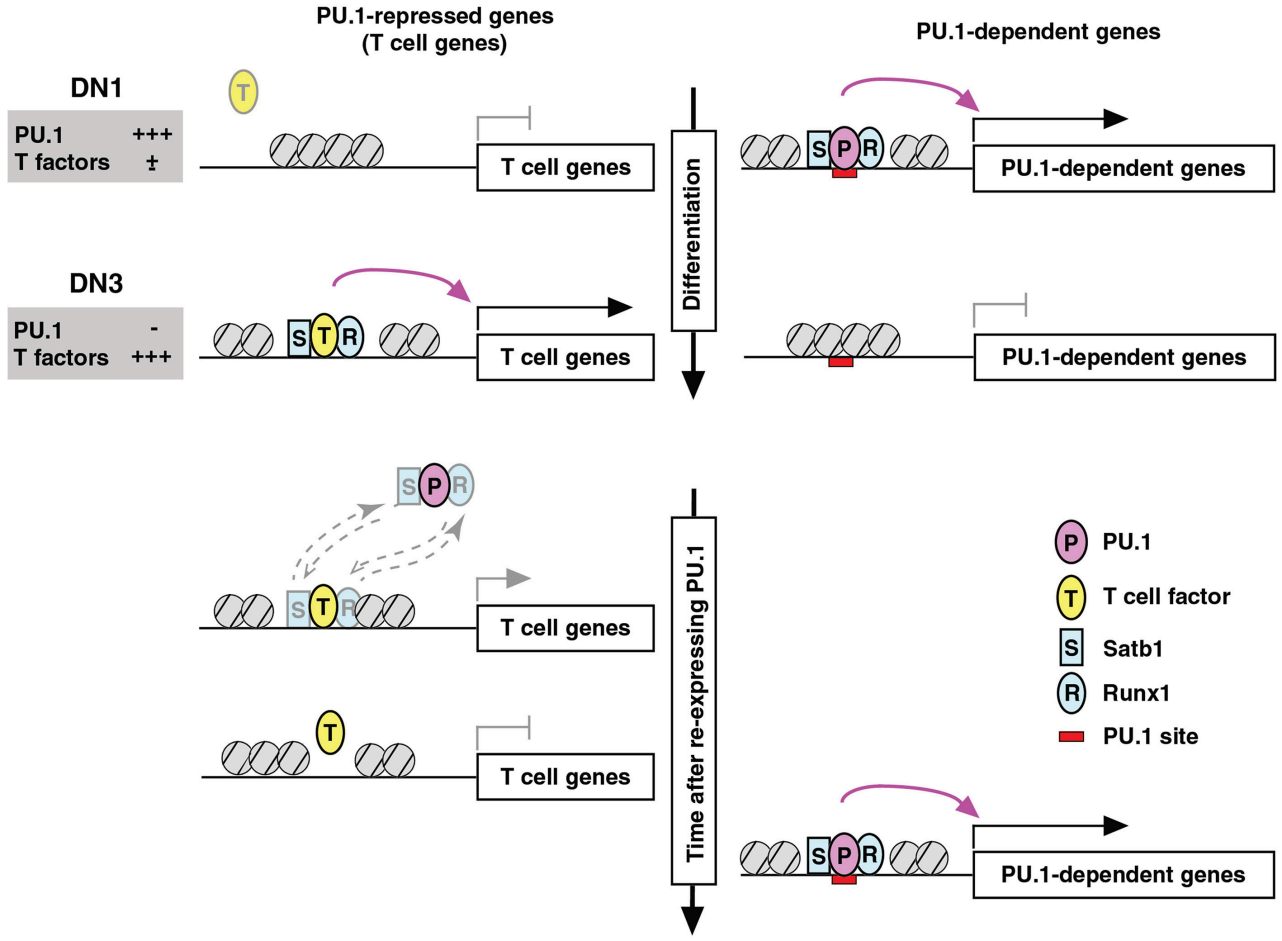
Repression by PU.1 can be caused by redistribution of a limiting co-regulator (co-regulator theft). Schematic depicts the complementary changes in factor binding patterns as well as in transcriptional activity of genes that are normally PU.1-dependent **(right)** or PU.1-inhibited **(left)**, across the developmental stages when PU.1 goes from high to low **(top)** and then in an experimental condition when PU.1 is re-introduced into committed cells that have already turned off endogenous PU.1 **(bottom)**. The figure shows that the redistribution of partner factors Runx1 (R) and Satb1 (S) by recruitment to PU.1 (P) binding sites occurs at the expense of sites that these factors would otherwise occupy together with other T-cell factors (T). Broken-line arrows **(lower left)** indicate that redistribution probably involves the dynamic equilibrium of binding of these factors between genomic sites that are differentially preferred in the presence and absence of PU.1. In at least some cases, the “theft” of the cofactors also results in relative closing of the chromatin at sites from which these cofactors are removed. Schematic modified from Hosokawa et al. (80).

PU.1 is a powerful organizer of the occupancy patterns of other transcription factors genome-wide. PU.1 binding shifts the disposition of other factors in the cell across the genome, even when their own expression levels and total numbers of binding sites remain essentially unchanged (80). The *positive* regulatory significance of these kinds of shifts is well established; many factors recruit others to collaborate with them in functional complexes at active enhancers [e.g., reviews by (17, 109–111)], and PU.1 is known to establish preferential binding sites for multiple other transcription factors in myeloid cells. However, in this case the positive impact is coupled with a *negative* regulatory consequence, via action at a distance (Figure 7). For PU.1 in pro-T cells, IRF and C/EBP family partners are mostly not available, but a key positive regulatory partner is Runx1 (previously known as AML1 or CBFα2), which has long been known to interact with PU.1 (and C/EBPα) to form a functional complex at its myeloid positive regulatory target sites (9, 112, 113). In pro-T cells, PU.1 binding sites in open chromatin genome-wide are highly enriched for Runx motifs, raising the possibility that Runx factors assist in the chromatin opening process (85), and proteomic analysis provides support for a strong representation of Runx1 in PU.1-containing complexes formed in the pro-T cell like Scid.adh.2C2 cell line (80). However, Runx1 also has sites at a large fraction of all enhancers active in the cells without PU.1 expression (Figure 7).

When epitope-tagged PU.1 was introduced into Scid.adh.2C2 cells and the complexes were isolated for proteomic analysis, much enrichment was seen for SWI/SNF complex components as well as some other chromatin modifiers (80). The preponderance of SWI/SNF complex interactions was consistent with the evidence that PU.1 usually acts as an activator. Based on the longstanding literature of PU.1–GATA factor antagonism through protein interaction (20–23), GATA-3 was expected to be present as well, and it was detectably enriched over background in these complexes. However, by far the most highly enriched sequence-specific transcription factor proteins interacting with PU.1 in these cells were Rest and Runx1 (80). Runx1 was of particular interest because of its sequence motif enrichment at PU.1 sites. Although Runx1 can act as a global chromatin accessibility organizer (114), PU.1 itself does not depend on Runx1 for establishing permissive sites for its binding, even in the “blank slate” context of the Scid.adh.2C2 cells (85). However, PU.1 strongly affected the sites where Runx1 bound, resulting in a dramatic shift in Runx1 binding site choices in tests of gain of PU.1 function (80). Supporting the physiological relevance of this mechanism, many of the same genomic sites where Runx1 was shifted by PU.1 in Scid.adh.2C2 cells underwent the reverse changes in Runx1 occupancy in normal primary pro-T cells, as they progressed from PU.1-high to PU.1-low developmental stages.

As expected, PU.1 recruits Runx1 to sites where Runx1 exerts measurable functional collaboration with PU.1, mostly to help in the positive regulation of PU.1 targets (80). However, the aspect of this redistribution that is most notable is that Runx1 is depleted in the process from alternative sites, and the sites that it abandons are themselves highly functional sites. The analysis is somewhat complicated by the fact that many developmentally important genes are linked with multiple Runx1 and/or PU.1 binding sites, only some of which gain or lose Runx1 occupancy. However, focusing on those genes that have Runx1 binding sites but not PU.1 binding sites, the genes that “lose” Runx1 binding when PU.1 is expressed clearly include a large fraction that depend quantitatively on Runx1 for their own expression. These genes show weak downregulation when Runx1 is disrupted by Cas9 and they show stronger downregulation when Runx1 is neutralized by a Runx1 dominant negative construct (80). Thus, the competition for Runx1 protein by PU.1 directly causes coupled positive and negative regulation, to cause a switch-like alteration in genome-wide cell state (Figure 7).

Three features of this mechanism are noteworthy (80). First, PU.1 does not appear to bind, even transiently, at the sites from which Runx1 is lost: Runx1 is competitively redistributed, but is not displaced. Thus, the PU.1 effect differs from “squelching” or other negative regulatory mechanisms where transcription factors are expelled by chromatin closing (115, 116). Second, one might expect that the Runx sites available for redistribution could have been vulnerable to dissociation because they were marginal quality binding sites in the first place; however, motif analysis shows that many of the Runx occupancy sites that are emptied when PU.1 is in the cell are high quality Runx sites in the upper half of the position weight matrix score distribution (80). Considered only as Runx sites, they are likely to be much higher affinity than the ones to which Runx1 moves, to occupy together with PU.1. Thus, the ternary (or higher-order) complexes nucleated by PU.1 are more favored binding sites for Runx1 when PU.1 is present than functionally relevant, high-quality Runx sites elsewhere. Finally, it is clear that this is a system-level mechanism. It is the limited pool of Runx1 operationally available for action across the genome that makes the impact of PU.1 a “zero-sum” outcome. Thus, the regulated level of Runx1 protein contributes to the switch-like impact made by the developmental shift from high-level PU.1 to PU.1 shutoff. However, given the high frequency of Runx factor utilization at multiple lymphoid enhancer sites, this kind of mechanism can propagate local PU.1 impacts to a much broader genomic scale.

The “theft” mechanism of repression by partner factor redistribution is not unique to the PU.1-Runx1 pair. PU.1 has an even stronger effect on binding site choice of Satb1, another transcription factor that is expressed throughout early T-cell development, and GATA-3 also shifts, when PU.1 is added, to occupy sites together with PU.1 (80). Although Satb1 in DN2-DN3 stages appears to have weaker effects on gene expression than Runx1, the PU.1-repressed genes that appear to be responding to Satb1 loss are different from those that are most dependent on Runx1, broadening the full impact of this mechanism (80). A very similar phenomenon has been reported earlier by Jenner and colleagues for the effect of T-bet on GATA-3 in establishing the Th1 cell program (117, 118). Thus, “partner factor theft” can be an integral part of the machinery for program choice operated by lineage-determining transcription factors.

## The PU.1 Regulome in Early pro-T cells and Its physiological Roles

### PU.1 Target Genes: Gene Network Roles and Developmental Timing

**Figure 8:**
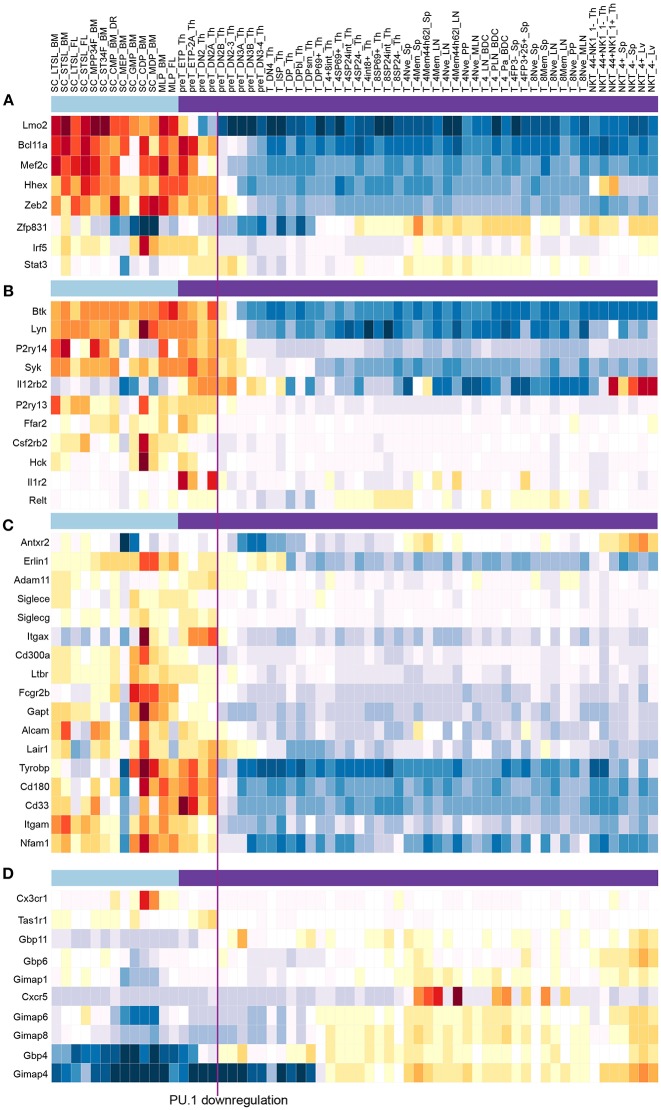
PU.1 globally regulates genes involved in multiple signaling and cell biological properties as well as other “phase 1” transcription factors across the stages when PU.1 is expressed. Summary of normal expression patterns of representative groups of PU.1 regulated genes is shown, illustrated using the ImmGen (119) (www.immgen.org) “My Gene Set” browser (http://rstats.immgen.org/MyGeneSet_New/index.html; Microarray V1). Natural levels of expression are shown in different “Stem and Progenitor” cell sets (120) (under light blue bar) and in successive stages of “αβ T cell” development (121) (under purple bar), where the color scale represents z score (warm colors, high expression; cold colors, low expression). Vertical line between “preT_DN2A_Th” and “preT_DN2B_Th” relates these stages to the timing of commitment, when PU.1 levels decline. **(A)** Genes encoding transcription factors activated by PU.1 in multiple tests (58, 79, 85). **(B)** Representative genes activated by PU.1 that encode tyrosine protein kinases, cytokine receptors, and G protein coupled receptors, from Table 1 [data from Ungerbäck et al. (85)]. **(C)** Genes activated by PU.1 that encode additional cell surface molecules, signaling receptors and adhesion molecules, from Table 1. **(D)** Genes repressed by PU.1, encoding chemokine receptors and G-protein coupled receptors, from Table 1. Functional clusters used in this summary were as defined by DAVID Gene Functional Classification tool (DAVID 6.8) (https://david.ncifcrf.gov/gene2gene.jsp).

While indirect regulation plays a large role in its developmental impact, the target genes that PU.1 directly regulates are ultimately crucial for understanding what this factor contributes to the T-cell program. PU.1's action as a positive regulator implies that most of its direct target genes should be expressed in a pattern concordant with its own expression. Indeed, PU.1-activated target genes are preferentially expressed in the earliest stages of T-cell development (examples shown in Figure 8). Many of them are expressed also in at least one of the other contexts where PU.1 is active: in myeloid and dendritic lineage cells, in B lineage cells, and particularly also in multipotent progenitor cells (Figure 8). Among the smaller number of genes that appear to be directly repressed by PU.1, most are specific for later stages of T-cell development. These patterns reinforce the case for PU.1's impact in shaping the developmental timecourse of gene expression in pro-T cells.

Developmentally potent transcription factors often transform a cell's identity by positively or negatively regulating the expression of other transcription factors. As noted above, PU.1 can have this effect on early pro-T cells when it is overexpressed and the cells switch to a non-T cell lineage program. But to what extent does PU.1 control the expression of other transcription factors within the T-cell program? It has become clear that the progression of cells through T-cell commitment involves the ordered downregulation of a substantial set of progenitor-specific transcription factors, called “Phase 1” factors in this context, concomitant with the upregulation of T-lineage affiliated factors (34, 65, 84, 121, 122). PU.1 itself is downregulated at stage when multiple other Phase 1 factors are downregulated, and a key question is whether the withdrawal of positive PU.1 input plays a role in the downregulation of these progenitor factors. With respect to PU.1-mediated repression, some T-cell factors are already upregulated while PU.1 is still highly expressed (GATA-3, TCF-1, and the Notch target Hes1), but others are upregulated only during the period when PU.1 declines (Bcl11b, Ets1, Lef1) and could, in principle, have their expression timing affected by PU.1 negative regulation. To what extent does PU.1 actually control these gene expression patterns under the circumstances of actual pro-T cell development, i.e. with strong Notch signaling that prevents lineage switching?

Data from multiple studies show that PU.1 regulates a subset of developmental control genes but is not alone in its actions. Varied PU.1 gain and loss of function perturbations in the DN2-DN3 stages show that PU.1 does provide positive input into a discrete subset of Phase 1 regulatory genes, with *Mef2c, Lmo2, Bcl11a*, and often also *Hhex* responding over a range of different tests (58, 79, 80, 85). Consistent with an evolutionarily conserved program in regulating these genes, the developmental expression patterns of these genes and PU.1 (*Spi1*) in human pro-T cells (Thy1—Thy4) are similar to their patterns in murine pro-T cells (35). PU.1 is not uniquely responsible for the Phase 1 gene expression pattern, however, for other Phase 1 genes are either unaffected or moderately inhibited by PU.1, as discussed elsewhere (79, 123). Additional factors also probably collaborate with PU.1 to fine-tune the responses of *Mef2c* and *Lmo2*, for they are already declining by the end of ETP stage, multiple cell cycles before DN2b stage when PU.1 itself declines (65, 67, 84, 121, 124) (Figure 8A). Thus, PU.1 is likely to be one of several important positive regulators for these genes. PU.1 can indeed have negative regulatory effects on some of the T-cell factors that are upregulated during commitment, but these effects are greatly limited when the analysis is confined to PU.1 activities within the T-cell program. *Hes1, Tcf12* (HEB), *Ets1*, and *Lef1* are strongly affected in cells making a lineage switch, but none of these are measurably repressed in cells within the T-cell path. The cell cycle-regulatory locus *E2f2*, which is also upregulated during commitment, is rare among transcription factor coding genes in that it does appear to be under active repression by PU.1 until the transition to commitment. Thus, within the T-cell pathway, PU.1 has a specific role in promoting maintenance of certain Phase 1 regulatory genes before commitment, but little role in repressing T-cell differentiation regulators directly.

### PU.1 as a Choreographer of Thymocyte Cell Biology

An important result from the genome-wide analysis of PU.1 target genes has been recognition of the major gene sets that it does actively control in early pro-T cells. The number of high confidence PU.1 target genes within the T-cell pathway that code for transcription factor genes is low (Figure 8A). In contrast, Gene Ontology and Pathway analyses as well as simple gene lists reveal that PU.1 directly controls major systems of cytokine receptors, chemokine receptors, tyrosine protein kinases, G-protein receptor signaling molecules, and adhesion or cytoskeletal system molecules (Table 2) (85). These directly regulated targets, some of them studied little, if at all, in T-cell development to date, might have a transformative impact on the cell biology of the developing lymphocytes between the stages when PU.1 is present and when it is shut off.

**Table 2:** Gene ontology and pathway classifications of genes regulated by PU.1 in pro-T cells.

**(A) Genes upregulated by PU.1 in CD25**^****+****^ **cells with gain of function, downregulated with sgRNA, relative to all genes expressed in cells**		
**GO biological process complete (top 21)**	**Fold enrichment**	**Adjusted *P*-value**
Peptidyl-tyrosine phosphorylation (GO:0018108)	7.18	2.40E-02
Myeloid leukocyte activation (GO:0002274)	7.05	9.14E-03
Reactive oxygen species metabolic process (GO:0072593)	6.97	3.05E-02
Regulated exocytosis (GO:0045055)	6.87	3.43E-02
Peptidyl-tyrosine modification (GO:0018212)	6.87	3.43E-02
Exocytosis (GO:0006887)	5.11	1.14E-02
Immune response-activating signal transduction (GO:0002757)	4.99	1.46E-02
Inflammatory response (GO:0006954)	4.76	7.00E-06
Immune response-regulating signaling pathway (GO:0002764)	4.74	2.53E-02
Activation of immune response (GO:0002253)	4.63	6.83E-03
Myeloid cell differentiation (GO:0030099)	4.55	3.80E-03
Positive regulation of protein secretion (GO:0050714)	4.21	2.11E-02
Regulation of body fluid levels (GO:0050878)	4.21	4.39E-02
Positive regulation of peptide secretion (GO:0002793)	4.15	1.23E-02
Adaptive immune response (GO:0002250)	4.1	2.92E-02
Positive regulation of defense response (GO:0031349)	4.07	1.59E-02
Regulation of MAP kinase activity (GO:0043405)	3.95	2.32E-02
Defense response to other organism (GO:0098542)	3.93	3.28E-03
Regulation of inflammatory response (GO:0050727)	3.87	2.96E-02
Immune effector process (GO:0002252)	3.74	5.31E-04
Innate immune response (GO:0045087)	3.73	3.02E-04
**(B) Genes downregulated by PU.1 in CD25**^**+**^ **cells with gain of function, upregulated with sgRNA, relative to all genes expressed in cells**		
**GO biological process complete**	**Fold enrichment**	**Adjusted** ***P*****-value**
Defense response to protozoan (GO:0042832)	27.62	1.72E-02
Response to protozoan (GO:0001562)	25.11	2.57E-02
Cell activation (GO:0001775)	4.44	2.43E-03
Immune response (GO:0006955)	3.55	6.41E-03
Immune system process (GO:0002376)	2.86	2.26E-04
Cellular response to stimulus (GO:0051716)	1.75	4.20E-02
**(C) Genes upregulated by PU.1 in CD44**^**+**^ **CD25- cells with gain of function, downregulated with sgRNA, relative to all genes expressed in cells**		
**GO biological process complete (top 21)**	**Fold enrichment**	**Adjusted** ***P*****-value**
Regulation of coagulation (GO:0050818)	8.85	5.55E-03
Regulation of blood coagulation (GO:0030193)	8.58	2.49E-02
Regulation of hemostasis (GO:1900046)	8.36	2.98E-02
Positive regulation of inflammatory response (GO:0050729)	7.17	3.76E-05
Myeloid leukocyte activation (GO:0002274)	6.03	1.61E-02
Regulated exocytosis (GO:0045055)	5.93	4.94E-02
Defense response to bacterium (GO:0042742)	5.16	5.18E-03
Positive regulation of stress-activated protein kinase signaling cascade (GO:0070304)	5.04	2.95E-03
Inflammatory response (GO:0006954)	4.98	5.37E-09
Positive regulation of stress-activated MAPK cascade (GO:0032874)	4.77	1.29E-02
Regulation of body fluid levels (GO:0050878)	4.62	1.78E-04
Leukocyte activation involved in immune response (GO:0002366)	4.53	4.90E-02
Positive regulation of defense response (GO:0031349)	4.51	2.71E-05
Positive regulation of MAP kinase activity (GO:0043406)	4.42	6.86E-03
Exocytosis (GO:0006887)	4.29	4.18E-02
Regulation of inflammatory response (GO:0050727)	4.29	6.51E-05
Positive regulation of response to external stimulus (GO:0032103)	4.09	2.38E-03
Regulation of MAP kinase activity (GO:0043405)	4.01	8.19E-04
Activation of immune response (GO:0002253)	3.85	3.77E-02
Positive regulation of protein serine/threonine kinase activity (GO:0071902)	3.8	1.28E-02
Defense response to other organism (GO:0098542)	3.7	8.48E-04
**(D) Genes downregulated by PU.1 in CD44**^**+**^ **CD25- cells with gain of function, upregulated with sgRNA, relative to all genes expressed in cells**		
**GO biological process complete**	**Fold enrichment**	**Adjusted** ***P*****-value**
T cell activation (GO:0042110)	4.77	3.82E-04
Lymphocyte activation (GO:0046649)	4.51	7.36E-07
Cell-cell adhesion (GO:0098609)	4.22	9.44E-03
Cell activation (GO:0001775)	4.19	1.78E-08
Lymphocyte differentiation (GO:0030098)	4.18	1.07E-02
Leukocyte activation (GO:0045321)	4.12	1.14E-06
Regulation of cell-cell adhesion (GO:0022407)	3.98	6.49E-04
Leukocyte differentiation (GO:0002521)	3.52	2.88E-02
Positive regulation of cell adhesion (GO:0045785)	3.45	2.10E-02
Regulation of defense response (GO:0031347)	3.11	1.15E-02
Regulation of cell adhesion (GO:0030155)	3.09	1.01E-03
Biological adhesion (GO:0022610)	2.9	2.29E-02
Positive regulation of transcription by RNA polymerase II (GO:0045944)	2.46	2.21E-03
Regulation of immune system process (GO:0002682)	2.35	3.85E-03
Immune system process (GO:0002376)	2.3	3.44E-05
Regulation of multicellular organismal process (GO:0051239)	1.74	3.15E-02
Positive regulation of biological process (GO:0048518)	1.48	6.54E-03

*The table shows PANTHER Overrepresentation Analysis (www.geneontology.org) of categories of genes upregulated or downregulated by PU.1. In each case, responding genes were defined by reciprocal changes in expression in PU.1 gain of function and PU.1 loss of function experiments in the DN2a-DN2b interval as in Table 1. Whereas Table 1 shows the three-way intersection of genes affected in loss of function, in gain of function for cells remaining CD25^+^, and in gain of function for cells becoming CD25^−^ CD44^+^, here the effects of the gain of function perturbations were separated to allow comparison of results from cells remaining in the T-cell pathway (CD25^+^) with results from cells likely deviating toward another fate (CD44^+^). Database for comparison was all genes expressed in control DN2 cells. Statistical results shown are for a Fisher Test with Bonferroni correction for multiple sample testing. The PANTHER Overrepresentation Test version was released 2018-10-10 using the GO Ontology database released 2018-10-08. For PU.1 activated genes, only the top 21 enriched categories are shown*.

Potentially important clues to PU.1 roles are the prominence among positively regulated PU.1 targets of genes encoding specific cytokine receptors not yet studied in T-cell biology (e.g., Pdgfrb); multiple protein tyrosine kinases (Btk, Syk, Hck, Lyn); and G-protein coupled receptors (Ffar2, P2ry13, P2ry14) and G protein signaling mediators (Gng2 and Rgs18); while PU.1 represses other G protein signaling mediators (Gimap and Gbp family members). In addition, PU.1 directly promotes expression of cell surface molecules (CD33, CD34, CD44) used as markers for stages in early T-cell development, but which *in vivo* work to mediate environmental interactions, and it drives expression of adhesion molecules (integrins and Siglecs) as well as cytoskeletal components such as Coro2a and Myo1f. Representative samples of the expression patterns of such genes are shown in Figures 8B–D. The result is that not only signaling capability but basic properties of adhesion, motility and chemoresponsiveness of the cells can be under PU.1 control in the early stages of T-cell development. While these effects are not seen as direct transcriptional regulation of other transcription factor coding loci, such target genes should have numerous impacts on activation pathways in the cells that induce transcriptional as well as migratory responses to environmental signals.

The PU.1-high stages of thymocyte development are relatively obscure in the context of the whole thymus, yet their accurate regulation is crucial for establishment of immune system homeostasis and avoidance of leukemia (125, 126). These stages span multiple cell cycles *in vivo* and *in vitro* (33, 39, 67, 124). However, cells in these stages are hard to visualize in the intact thymus, as only a few cells per day are granted regulated entry into the thymic antechamber (127), then migrate slowly through the cortex, dispersing among a vast excess of more advanced T-cell precursors, as they begin to differentiate toward commitment (128). In postnatal mice, the entry point is thought to be formed by specialized endothelial cells at the cortical/medullary border of the thymus (129). Following an unknown triggering signal, after a variable delay (125), the cells in each cohort then begin to migrate centrifugally toward the outer thymic cortex, and cell surface marker expression patterns imply that it is somewhere midway in the course of this migration that the individual cells undergo lineage commitment [reviewed by (60, 130)]. Because of the extreme rarity of these very immature cells relative to the later-stage thymocytes at any given time, they were almost impossible to study in depth before the development of *in vitro* culture systems (88), which have continued to be informative to the present. However, the types of genes positively regulated by PU.1 are overwhelmingly in categories likely to be involved in mediating the interaction of the cells with very specific environments. The tests of PU.1 function in these early pro-T cells that have been done so far present the cells with Notch ligands and cytokines, but could be fundamentally lacking in other molecules presented by the normal thymic environment. It will be of great interest to discover which anatomical subdomains of the thymus actually supply the molecules that interact with the potentially important receptors and adhesion molecules that PU.1 enables the cells to express, and what responses they trigger in these earliest T-cell precursors.

## PU.1 and the Regulation of Lymphoid Development in Ontogeny

The studies reviewed throughout this paper have characterized the roles of PU.1 in T-cell development in the young postnatal mouse or in late fetal life. Very recent work has now placed these roles of PU.1 into a wider developmental perspective.

The cells used for *in vitro* differentiation as well as *in vivo* analysis in the work reviewed above have all been derived from waves of hematopoiesis that begin with definitive hematopoietic stem cells, which first appear in the mouse fetal liver by about day 11.5 of gestation and may be followed by additional stem-cell waves through the end of gestation (day 20) (131). Thus, postnatal thymocytes and any *in vitro* differentiation cultures seeded with cells from bone marrow or fetal liver from E15 onward are likely to come from true stem cell origins. However, there are earlier hematopoietic progenitors in the embryo that derive from yolk sac, cells with varied developmental potentials but without true stem-cell self-renewal. The first wave of T cell development in the fetus is now thought to arise from these non-stem-cell precursors in the yolk sac (132, 133). It has long been recognized that the earliest fetal thymocytes are different from later waves of developing thymocytes in terms of their abilities to generate particular classes of TCRγδ cells (134) and in terms of their extremely fast differentiation kinetics, both *in vivo* and in fetal thymic organ culture or stromal coculture systems (104, 133, 135). This is now understood to be intrinsically programmed (136) and due to an altered pathway of differentiation in the first-wave cells, which results in T-cell lineage commitment even before entry into the thymus (137–139).

Remarkable differences have been reported between genetic requirements for T cell development derived from earlier and later waves of prethymic progenitors. For example, the crucial T-lineage transcription factor TCF-1, which plays roles in numerous phases of thymocyte development (95, 140–145), is essential to maintain adult T-cell production but dispensable in fetal and early postnatal T cell development (146). A wave of fetal T cell development can also, apparently, be generated without PU.1 (71). In the case of PU.1, the change in its role occurs within fetal life, and this has now been sharply situated in the transition from precociously committed “first-wave” precursors to precursors that enter the thymus while still multipotent (147). ETPs derived from these precursors naturally express lower levels of PU.1 than adult ETPs, but they are almost unchanged in their ability to generate early fetal T cells when the level of PU.1 is reduced still further (~5 fold) by deletion of the major upstream regulatory element of PU.1 (147). In contrast, the same five-fold diminished level of PU.1 sharply degrades the ability of later fetal hematopoietic stem and progenitor cells to generate T cells at all, *in vivo* or *in vitro*, with functional and phenotypic defects evident in the mutants in both multipotent progenitors and newly-entered intrathymic ETPs, as early as in the late fetus. This difference in PU.1 dependence accompanies a subtly different T-cell developmental program. Gene expression differences have been noted between the normal first-wave fetal and adult pro-T cells in the thymus at corresponding stages which indicate that the fetal program drives accelerated development (148, 149). Montecino-Rodriguez et al. point out that these differences conspicuously include reduced initial expression of multiple PU.1-dependent genes in the fetal cells (147). Thus, not only is the first-wave fetal program less dependent on PU.1, but also it may rely on relatively low PU.1 activity for its very distinctiveness. These results therefore support a role for PU.1 in delaying differentiation in order to allow more extended proliferation before commitment, showing how the importance of this role is ontogenically scaled to the needs of the developing organism.

The first-wave precursor cells, also uniquely, enter the thymus by a different route than all subsequent waves. Instead of entering through the blood vessels near the cortical-medullary junction, these early cells migrate through cervical-region mesenchyme to the thymic anlage before it is vascularized. The thymus does not yet have a capsule to present a physical barrier, and the first-wave cells enter directly through the future outer cortex. Thus, they may not use the same interactions with basement membrane, endothelial cells, or chemokine gradients as any future wave of thymic precursors. Not only are these cells intrinsically programmed to cut short the stages supported by PU.1-dependent transcriptional regulators, but also they can dispense with many of the cell biological tools that PU.1 may provide to later-wave successors to navigate the adult or late-fetal thymus.

## Conclusions and Future Questions

PU.1 is a broad regulator of the properties of the cells that first enter the thymus, and it helps to determine their proliferation and rate of progression to commitment after they arrive. While dysregulated PU.1 can cause trans-differentiation to myeloid or dendritic-cell fates, endogenous PU.1 normally plays a protracted role within early T-cell development. Its target genes are occasionally repressed but mostly activated by PU.1 binding, and they confer on the cells distinctive stage-specific transcription factor expression patterns as well as a rich array of stage-specific cell biological features that await proper functional analysis. This positive regulatory role is one result of PU.1's strong pattern of binding across the genome, its prominent occupancy of open chromatin sites, and the evidence that it helps to maintain the open chromatin states at bound regulatory sites as long as it is expressed. The number of genes that respond quickly to changes in PU.1 activity may only account for a minority of all the genomic sites where PU.1 is found engaged; at other sites, its role could be structural or redundant with other factors. However, it is clear that PU.1 also affects the activity of certain genes that it does not bind to directly, via creating preferential interaction sites for other factors that can deplete the regulatory elements of those factors' alternative target genes. Through chromatin state placeholding and “coregulator theft” as well as through its own direct transcriptional activities, PU.1 pervades the regulatory state of early T cells as long as it is expressed.

This phase comes to an end when other transcription factors finally accumulate to the point where they can shut PU.1 off. The best current candidates for this silencing activity include GATA-3 (104, 105), TCF-1 or LEF-1 (37), and especially Runx1 (80, 150–152), probably working in a dose-dependent combination, although the mechanism through which they finally achieve the ability to repress PU.1 has not yet been reported. Importantly, the duration of the PU.1 activity phase is regulated to vary among different ontogenic waves of T cell development. It probably extends for over 10 days for the thymocytes in young adult mice (153), where it is crucial for successful T-cell generation (2, 147). In contrast, for many first-wave fetal thymocytes it may last only a day or two, and is mostly or entirely dispensable (71, 147). This indicates that the specific constellation of functions that PU.1 serves in T-cell development is a module within the larger T-cell developmental program that can be deployed optionally to serve a particular role. Perhaps it is more important for scaling the population dynamics of T-cell production as the animal finishes gestation and grows, or for promoting accurate migration through distinct thymic microenvironments, than for making T cell precursors *per se*.

The pioneering role of PU.1 on the genome raises fascinating questions for future study that connect mechanism with developmental lineage selection. Hematopoietic progenitors express PU.1 before they enter the thymus, but the pattern of its occupancy is not well defined at that stage, so the onset of PU.1's pioneering activity in precursors that will eventually generate T cells is not easy to study. The mechanisms discussed in this review show that it establishes a pre-pattern that can influence the binding of the other transcription factors expressed in the cell throughout multiple cell cycles in the thymus. It is not clear, though, how this particular pre-pattern is set, to be distinguished from PU.1 binding patterns in B cells and myeloid cells (84). The question could be linked with the deeper mystery of the factors involved in designating some multipotent precursors to enter the thymus in the first place, as opposed to remaining in the bone marrow for programming into B cells, natural killer cells, or innate lymphoid cells. So far the innate lymphoid cell developmental program in particular appears to resemble the intrathymic T-cell program in many respects (154–157), enough to raise the question of what makes T-cell precursors wait to activate genes like *Tcf7* and *Gata3* until they reach the thymus. Is PU.1 part of the answer? The system-wide impact of PU.1 on other factors suggests that in scenarios where PU.1 is absent, the same T-cell transcription factors might initially choose different binding sites. Indeed, pro-T cells that have PU.1 acutely deleted at an early stage do not only differentiate faster along the T lineage; they also tend to shift to a natural killer-like program more readily than controls (79). Thus, activity of PU.1 may be important, also, to block certain alternative differentiation paths for pro-T cells. In the end, is T-cell lineage fidelity itself partly a legacy of PU.1's transient role?

## Author Contributions

ER wrote the paper, contributed to ideas in the review, and directed research that led to this review. HH and JU carried out research that led to this review, contributed to ideas in the review, provided some figures and edited the paper.

### Conflict of Interest Statement

The authors declare that the research was conducted in the absence of any commercial or financial relationships that could be construed as a potential conflict of interest.
